# Comparative Study on the Fermentation Characteristics of Selective *Lactic Acid* Bacteria in Shanxi Aged Vinegar: Pure Culture Versus Co-Culture

**DOI:** 10.3390/foods13213374

**Published:** 2024-10-23

**Authors:** Qi Li, Yujing Zhang, Chaomin Wang, Xiaoyu Zhang, Ruteng Wei, Yunlong Li, Qiqiong Li, Nv Xu

**Affiliations:** 1 Shanxi Province Vinegar Brewing Technology Innovation Center, College of Food Science and Engineering, Shanxi Agricultural University, Jinzhong 030801, China; sxauliqi1992@outlook.com (Q.L.); 15369402069@163.com (Y.Z.); wangchaomin2019@163.com (C.W.); xiaoyuzhang2005@163.com (X.Z.); weiruteng@sxau.edu.cn (R.W.); 2 Shanxi Institute for Functional Food, Shanxi Agricultural University, Taiyuan 030031, China; liyunlong125@126.com; 3 State Key Laboratory of Food Science and Resources, Nanchang University, 235 Nanjing East Road, Nanchang 330047, China; liqq7@ncu.edu.cn

**Keywords:** Shanxi aged vinegar, microbial diversity, *lactic acid* bacteria, acetic fermentation

## Abstract

The diversity of the microbial community structure plays a crucial role in the flavor and nutritional value of Shanxi aged vinegar in fermentation. Illumina Miseq high-throughput sequencing identified thirteen bacterial genera, with *Lactobacillales* (44.89%) and *Acetobacter* (21.04%) being the predominant species. Meanwhile, the fermentation characteristics of selected *lactic acid* bacteria strains isolated from Shanxi aged vinegar were studied in different media. The results showed that the biomass, and physical and chemical indices, as well as flavor compounds of the four strains of *lactic acid* bacteria in the simulated vinegar fermented grains medium were superior to those in barley and pea medium and sorghum juice medium. The bacterial interaction was conducted to investigate the effects on growth, the physicochemical indices, and flavor substances in order to determine the optimal combination. Furthermore, the interaction between pure cultures and co-cultures of *lactic acid* bacteria in a simulated vinegar culture medium was investigated, with a focus on the impact of this interaction on strain growth, fermentation characteristics, and flavor compound production. Compared with the pure culture, when strains L7 and L729 were co-inoculated, the reducing sugar content was 0.31 ± 0.01 g/100 g, total acid content was 3.02 ± 0.06 g/100 g, acetoin content was 2.41 ± 0.07 g/100 g, and total organic acid content was 3.77 ± 0.17 g/100 g. In terms of flavor compounds, the combined culture system exhibited higher levels of esters, aldehydes, and acids compared to pure cultures or other co-culture systems. This study revealed the fermentation characteristics of selected *lactic acid* strains in Shanxi aged vinegar under different conditions and their interaction in simulated vinegar fermentation media, which could provide theoretical support for the safety and health evaluation of aged vinegar.

## 1. Introduction

Vinegar has been produced for over 3000 years worldwide and can be utilized both as an acidic condiment and a functional food [1]. Vinegar brewing is a process that involves the interaction of various microorganisms, primarily *lactic acid* bacteria, yeasts, acetic acid bacteria, and moulds [2]. During fermentation, a variety of organic acids, amino acids, alcohols, esters, and other aromatic components are produced [3,4]. *Lactic acid* bacteria are a type of bacteria that can enhance the flavor of vinegar during fermentation [5]. *Lactic acid* bacteria belong to a group of bacteria that can convert sugars into *lactic acid* during the vinegar fermentation process, enhancing the vinegar flavor. They also produce non-volatile organic acids (such as malic acid and palmitic acid) through *lactic acid* fermentation, adjusting the sour taste in vinegar and making it taste smoother [3,6]. For humans, most *lactic acid* bacteria are beneficial as they can effectively enhance the body’s immune function [7,8]. Many researchers have isolated various functional *lactic acid* bacteria from vinegar, which has also contributed to the advancement of vinegar production [9]. The benefits of edible vinegar mainly come from the metabolites present in the product. However, the presence of *lactic acid* bacteria as probiotics can also provide additional benefits [10].

Based on different brewing techniques, vinegar can be classified into two categories: liquid-fermented vinegar and solid-fermented vinegar [11,12]. *Lactic acid* bacteria are primarily involved in the production of organic acids and volatile aroma compounds during solid-state vinegar fermentation. *Lactic acid* bacteria primarily produce *lactic acid* and various organic acids through three metabolic pathways: homolactic fermentation, obligatory heterolactic fermentation, and facultative heterolactic fermentation [9,10,13]. The *lactic acid*, succinic acid, and other non-volatile acids produced as a result of these processes can mitigate the sour taste caused by acetic acid and enhance the flavor. Additionally, they can also undergo esterification with alcohols and other substances to form different ester compounds that contribute to a vinegar aroma [14,15]. During traditional vinegar fermentation, *lactic acid* bacteria can produce a variety of organic acids, such as *lactic acid*, malic acid, and citric acid, as well as various amino acids that give vinegar its unique taste. *Lactic acid* bacteria can produce flavor compounds like ethylene oxide and 2,3-butanediol, along with functional components such as exopolysaccharides. In addition, during the fermentation process, there is interdependence, mutual preference, mutual competition, and mutual parasitism among microorganisms, which can affect their growth [16]. Therefore, understanding the growth law and metabolic characteristics of *lactic acid* bacteria can provide a theoretical basis for enhancing the acetic acid fermentation process of Shanxi aged vinegar and serve as a strain foundation for its quality.

Shanxi aged vinegar is mainly produced through the fermentation of raw materials such as sorghum, bran, rice husk, and grain bran that contain starch and sugar. The metabolic action of microorganisms in nature produces numerous catalytic enzymes to facilitate the conversion of acetic acid and other main components of vinegar [17]. Many researchers have combined pure culture and non-culture techniques to analyze the microbial community structure, succession patterns, and functional roles in Shanxi aged vinegar. These microorganisms do not act exclusively at a certain stage but rather become dominant at a particular stage, controlling the fermentation process in an orderly manner [3,4]. Numerous researchers used the traditional counting culture method and high-throughput sequencing method to study the microorganisms in Shanxi aged vinegar during fermentation. After analyzing the biological changes, various strains were finally screened and identified, including *Lactobacillus plantarum*, *Acetobacter pasteurella*, *Pediococcus lactis*, *Pediococcus pentose*, *Lactobacillus* JL6, and *Lactobacillus casei* CL21 [18,19]. Shanxi aged vinegar is a traditional solid-state fermentation process. In an open environment, microorganisms continue to enrich, grow together, and interact with each other, ultimately forming a vinegar product with a unique flavor. However, the flavor is produced through processes such as alcohol fermentation and acetic acid fermentation [9]. The current research mainly focuses on the flavor substances during the aging process of Shanxi aged vinegar, with little research conducted on the flavor compounds during its fermentation process. 

The production process of Shanxi aged vinegar involves naturally enriched multi-species co-fermentation. Therefore, this study utilized metagenomic high-throughput sequencing technology to investigate the microbial community structure of Shanxi aged vinegar, revealing the diversity of microorganisms involved in the process and identifying the dominant bacteria and succession patterns at different fermentation stages. In our previous stage, thirty strains of *lactic acid* bacteria were isolated and purified in our laboratory, out of which four strains (*Lactobacillus plantarum* SAVndL7 (L7), *Lactobacillus plantarum* SAVndL19 (L19), *Pediococcus acidilactici* SAVndL729 (L729), and *Pediococcus pentosaceus* SAVndL2422 (L2422) were screened out. This study systematically investigates the fermentation characteristics of these four *lactic acid* bacteria in different culture media as well as strain interactions with each other to provide a strain basis for ensuring the quality of Shanxi aged vinegar. 

## 2. Materials and Methods

### 2.1. Materials

#### 2.1.1. Samples

The samples of vinegar were collected from Zi Lin Vinegar Industry Co., Ltd., (Taiyuan, China), during the alcohol fermentation stage (J0, J1, J2, J4, J6, J8, J10, J12, J14, and J16) and the acetic acid fermentation stage (C0, C1, C2, C4, C6, C8, and C10) in two batches. The second batch of samples (#1 and #2) were collected two weeks apart and stored at −80 °C in a freezer for subsequent experiments. *Lactobacillus plantarum* SAVndL7 (L7), *Lactobacillus plantarum* SAVndL19 (L19), *Pediococcus acidilactici* SAVndL729 (L729), and *Pediococcus pentosaceus* SAVndL2422 (L2422) were isolated from the fermentation residue of Shanxi aged vinegar and preserved in the biological engineering laboratory of Shanxi Agricultural University.

#### 2.1.2. Chemical Reagents

Sodium hydroxide and glucose were purchased from Chemical Reagents Co., (Tianjin, China), State Pharmaceutical Import & Export Corporation. Oxalic acid, tartaric acid, pyruvic acid, malic acid, *lactic acid*, acetic acid, citric acid, succinic acid, etc. were obtained from Tianjin Jindong Tianzheng Fine Chemical Reagent Factory (Tianjing, China). 4-Methyl-2-pentanol was sourced from Shanghai Peco Reagent Research Institute (Shanghai, China). Agarose Gnd10 was acquired from Biowest in Spain (Shanghai, China). λHindⅢ DNA Marker and RNase A were purchased from Shanghai Shenggong Bioengineering Co., Ltd., (Shanghai, China). Cetyltrimethylammonium bromide (CTAB) was obtained from Fuzhou Feijing Biotechnology Co., Ltd., (Fujian, China). Cross-linked polyvinylpyrrolidone (PVPP) was procured from Chemical Reagents Co., State Pharmaceutical Import & Export Corporation. Sodium dodecyl sulfate (SDS) was sourced from Beijing Solarbio Technology Co., Ltd., (Beijing, China). The QIA quick PCR Purification Kit, QIA quick Gel Extraction Kit, TruSeq PE Cluster Kit, and End RepairMix were all purchased from Qiagen in Hilden, Germany. Trisnd balanced phenol, and Tris were also obtained from Beijing Solarbio Technology Co., Ltd. Ethylenediaminetetraacetic acid disodium salt (EDTA-Na) was procured through the Chemical Reagents Co., State Pharmaceutical Import & Export Corporation’s analytical reagent grade.

### 2.2. Methods

#### 2.2.1. Enumeration and Cultivation Techniques of *Lactic Acid* Bacteria

Each sample was added to 255 mL of physiological saline solution and mixed uniformly at 200× *g* on a shaker for 5 min. The resulting suspension was then diluted continuously with sterile physiological saline solution, and the bacterial and *lactic acid* bacteria counts were determined separately using Plate Count Agar (PCA) (incubated at 37 °C for 1–2 days) and Modified MRSMedium Base (MRS) culture media (incubated at 37 °C for 2–3 days).

The MRS culture medium contains 50 mL in a triangular bottle. Single colonies of L7, L19, L729, and L2422 were individually transferred culture medium using a sterile loop. The cultures were then incubated at 37 °C for 1–2 days to reach a bacterial concentration of 1 × 10^6^ CFU/mL, followed by cooling to 4 °C and centrifugation (8000× *g*) for 5 min. After discarding the supernatant, the bacterial suspension was suspended with sterile distilled water. Microscopic examination was conducted to adjust the cell concentration for each inoculation. Subsequently, the bacterial suspension was added to barley pea, maize juice, and simulated pickle media at a weight ratio of 2% and incubated at 37 °C in a constant temperature incubator for 24 h.

#### 2.2.2. Biomass Measurement Methods

Approximately 25 mL of samples were collected from each culture medium on days 2, 4, and 6. The samples were added to 225 mL of sterile physiological saline containing glass beads and thoroughly mixed at 140× *g* at room temperature for 30 min. Gradient dilution was used, and 100 µL of each diluted sample were spread on an MRS plate culture medium. Then, these were incubated at 37 °C for 48 h. Plates with a colony count ranging between 30–300 was selected, and *lactic acid* bacteria were identified based on colony morphology [20].

#### 2.2.3. Physical and Chemical Index Determination Method

(1) Determination of total acid, reducing sugar, total esters, and acetoin

The total acid content of the fermentation liquid was determined through titration with sodium hydroxide (1 mol/L, National Standards of the People’s Republic of China GB/T 12456–2021) [21]; the reducing sugar content was measured using Fehling’s titration method [22]; the total ester content was assessed using the sodium hydroxide saponification method (National Standards of the People’s Republic of China GB/T 19777-2005) [22]; and the acetoin content was determined with reference to the method of Chen et al. [23].

(2) Organic acid

About 5 mL of the fermentation broth was diluted with 50 mL of ultrapure water. The mixture was centrifuged at 12,000× *g* for 5 min, followed by filtration of the supernatant through a 0.22 μm microfiltration membrane. Subsequently, an external standard method was used to determine the concentrations of eight organic acids (oxalic acid, tartaric acid, pyruvic acid, malic acid, *lactic acid*, acetic acid, citric acid, and succinic acid) using chromatographic conditions including an Ultimate 3000 liquid chromatography system (Thermo Fisher scientific, Waltham, MA, USA); C18 4.6 × 150 mm column with a particle size of 5 μm; mobile phase consisting of 20 mmol/L NaH_2_PO_4_ at pH = 2.7; injection volume set at 20 μL; flow rate maintained at 0.8 mL/min; detection wavelength set at UV–Vis absorbance at a wavelength of 210 nm; and column temperature kept constant at room temperature [24].

(3) Determination of volatile aroma compounds

The determination of volatile aromatic compounds is conducted using gas chromatography—mass spectrometry (Agilent 6890N-5973MSD, Santa Clara, CA, USA). Approximately 5 mL of fermentation broth from each culture was extracted using headspace solid-phase microextraction (HS-SPME) with a 50 μL syringe. The mixture was prepared by adding 1 g of sodium chloride and 10 μL of internal standard (0.8775 g/L, methyl octanoate), followed by equilibration at a temperature of 40 °C for a duration of 15 min. The types and concentrations of volatile aroma compounds were determined through direct internal standardization. The chromatographic conditions were as follows: a VFnd5MS column (30 × 0.25 mm × 0.25 mm, Thermo Fisher scientific, Waltham, MA, USA), helium carrier gas at 99.999% purity, a flow rate of 1 mL/min, and no splitting used. The programmed temperature started at 40 °C for 3 min, then increased at a rate of 4 °C/min to reach and maintain 160 °C for 1 min, followed by an increase at a rate of 10 °C/min to reach and maintain 270 °C for another minute. Mass spectrometric conditions included an interface temperature of 280 °C, ion source temperature also at 280 °C, electron energy set at 70 eV, and scanned mass range from 41 to 500 amu [25]. The volatiles were compared to mass spectra and characterized using the NIST 17.0 mass spectrum library, achieving an 80% similarity.

#### 2.2.4. Sample Preparation, DNA Fragment Amplification, and Miseq Library Establishment

The weighed 3 g samples were placed in a pre-cooled mortar at −20 °C and ground to a fine powder using liquid nitrogen. The resulting powder was then transferred to a 2 mL centrifuge tube. Then, 2 mL of extraction buffer (containing 1 g/100 mL CTAB, 2 g/100 mL PVPP, 8.78 g/100 mL NaCl, 3.73 g/100 mL EDTA-Na, and 1.21 g/100 mL Tris) in mol/L phosphate buffer was added at 37 °C with agitation at 200× *g* for 30 min. Subsequently, the mixture was incubated with 200 μL of SDS (10 g/100 mL) in a water bath at 65 °C for 2 h while being agitated at 12,000× *g*. After low-temperature centrifugation for 15 min, the supernatant was removed and cooled to 4 °C. An equal volume of phenol–chloroform–isoamyl alcohol mixture (V/V/V = 25:24:1) was added and centrifuged again at 12,000× *g* for 20 min until the intermediate protein layer disappeared after repeated washing steps (3–4 times). Then, an equal volume of chloroform and isoamyl alcohol mixture (V/V = 24:1) was added, followed by low-temperature centrifugation at 12,000× *g* for 15 min. The precipitate that was obtained after the addition of 0.7 times the volume of isopropanol was further precipitated at −20 °C for 4 h under high-speed centrifugation conditions (12,000× *g*), followed by removal of the precipitate through low-temperature centrifugation for 15 min. The resulting precipitate was thoroughly rinsed with pre-cooled 70% ethanol before being dissolved in 80 μL ddH_2_O after complete evaporation of ethanol volatiles had occurred at room temperature. Finally, 5 μL of RNaseA solution (1 mg/mL) was added and incubated in a water bath at 37 °C for 20 min. Specific primers (bacteria: 515F-806R, PCR Master Mix) were added to the extracted metagenomic DNA as a template to amplify the V4 region of bacterial 16S rDNA, and expanded segments with a size of 200 bp from left to right were obtained. The corresponding region-specific primers were as follows: bacteria 515 F: 5′-GTGCCAGCMGCCGCGGTAA-3′ and 806 R: 5′-GGACTACHVGGGTWTCTAA-3′. The QIA quick PCR Purification Kit was used to purify the PCR products by adding End RepairMix and reacting at 20 °C for 30 min. DNA products qualified for electrophoretic detection were recovered using the QIA quick Gel Extraction Kit. The amplified DNA sequence length was detected with an Agilent 2100 bioanalyzer, and products with a sequence length ≥ 10,000 bp were recovered. The library was amplified using the TruSeq PE Cluster Kit, and the amplified library was sequenced on the MiSeq 2000 sequencing platform [23].

#### 2.2.5. Interaction of Different Combinations of *Lactic Acid* Bacteria

Four strains of *lactic acid* bacteria were activated, and single colony was inoculated into the MRS broth medium, followed by cultivation at 37 °C for 1–2 days to achieve a final concentration of 1 × 10^8^ CFU/mL. The cultured bacterial solution was inoculated into a 50 mL triangular bottle containing simulated vinegar culture medium (fermented vinegar grains from Shanxi aged vinegar factory were collected on the second day of acetic acid fermentation and loaded into a 250 mL conical flask—the flask was then sealed with four layers of gauze, creating a simulated fermented vinegar culture medium) with an inoculating amount of 4%, and incubated at 37 °C for a duration of 6 days. The biomass of L7, L19, L729, and L2422 was measured after 2, 4, and 6 days of fermentation. After the fermentation period, the basic physicochemical properties and flavor substances were measured. Additionally, the interactions among L7, L19, L729, and L2422 were studied.

### 2.3. Data Analysis

An independent t-test was used for statistical analysis. All data are presented as mean ± standard deviation (SD) with a sample size of 6–10, using GraphPad Prism (version 8.0.2, GraphPad Software, CA, USA). Each experiment was repeated three times. Capital letters represent variations among different strains in the same medium, while lowercase letters represent variations of the same strain in different media.

## 3. Results and Discussion

### 3.1. Dynamic Changes in Lactic Acid Bacteria Biomass During Alcohol and Acetic Acid Fermentation Stages

**Figure 1:**
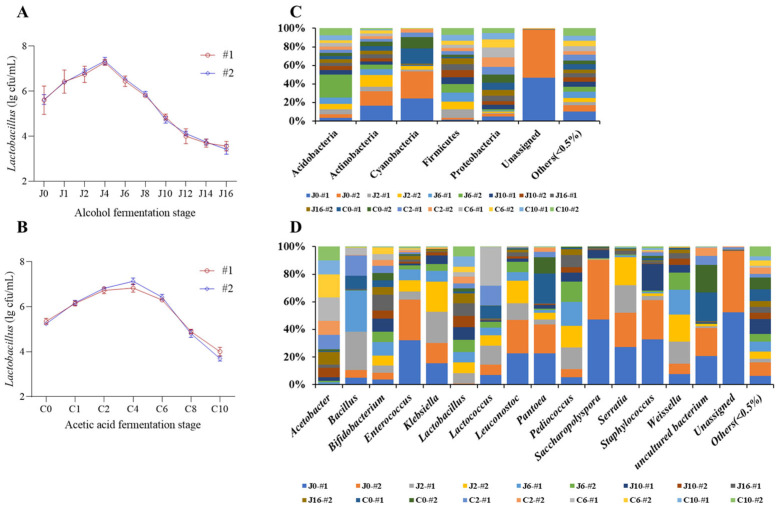
Dynamic changes in *lactic acid* bacteria biomass at the phylum and genus level in different batches of Shanxi aged vinegar during alcohol and acetic acid fermentation stages. Notes: #1 is the first batch of secondary samples; #2 is the second batch sample. (**A**) Traditional handmade two batches of alcohol fermentation stage *lactic acid* bacteria biomass. (**B**) Traditional handmade two batches of acetic acid fermentation stage *lactic acid* bacteria biomass. (**C**) Dynamic changes in bacterial phylum level during the fermentation process of aged vinegar. (**D**) Dynamic changes in bacterial genus level during the fermentation process of aged vinegar.

As shown in Figure 1A,B, the biomass of *lactic acid* bacteria increased initially and then decreased in the alcohol and acetic acid stages under the traditional manual mode. During the alcoholic fermentation stage, there was a significant upward trend in the biomass of *lactic acid* bacteria for both batches from day 0 to day 4, reaching their maximum values on day 4 at 7.28 ± 0.13 log CFU/mL and 7.34 ± 0.16 log CFU/mL, respectively. The biomass of *lactic acid* bacteria gradually decreased with fermentation time, reaching its minimum at the end of alcohol fermentation at 3.56 ± 0.21 log CFU/mL and 3.42 ± 0.19 log CFU/mL, respectively. During the acetic acid fermentation stage, there was a significant increased trend in the biomass of *lactic acid* bacteria for both batches from day 0 to day 4 of acetic acid fermentation, reaching their maximum on day four at 6.83 ± 0.18 log CFU /mL and 7.13 ± 0.15 log CFU /mL respectively. The biomass of *lactic acid* bacteria gradually decreased with the fermentation time, and reached the minimum at the end of acetic acid fermentation, which was 4.00 ± 0.19 log CFU/mL and 3.70 ± 0.11 log CFU/mL, respectively. The trend of *lactic acid* bacteria biomass during the alcohol and acetic acid fermentation stages was not significant across batches, indicating batch stability.

### 3.2. Dynamic Changes in Bacterial Flora Structure During the Traditional Fermentation Process of Shanxi Aged Vinegar

As shown in Figure 1C, the main bacterial phyla in traditional fermentation are as follows: Firmicutes (52.49%), Proteobacteria (33.66%), Cyanobacteria (11.43%), Actinobacteria (1.03%), and Acidobacteria (0.60%). In traditional fermentation, the relative abundances of Firmicutes and Proteobacteria were relatively large throughout the entire aged vinegar fermentation process. Among them, the relative abundance of Firmicutes at the alcohol fermentation stage was greater than that at the acetic acid fermentation stage, and it reached a maximum of 90.97% on the sixth day of alcohol fermentation; it was the dominant microorganism in traditional fermentation. The relative abundance of Proteobacteria showed an upward and then downward trend, reaching its maximum value of 65.88% on the sixth day of acetic acid fermentation. In the alcohol fermentation stage, the relative abundance of Cyanobacteria was relatively large, and its relative abundance reached 60.26% on the zeroth day of acetic acid fermentation. In the entire fermentation process, Actinobacteria and Acidobacteria were detected, and their relative abundance decreased as the fermentation time increased. As shown in Figure 1D, the main bacterial genus in traditional fermentation was as follows: *Lactobacillus* (44.89%), *Acetobacter* (21.04%), *Pediococcus* (4.72%), *Weissella* (2.21%), *Bacillus* (1.06%), *Pantoea* (0.61%), *Bifidobacterium* (0.55%), *Leuconostoc* (0.41%), *Klebsiella* (0.34%), *Saccharopolyspora* (0.19%), *Staphylococcus* (0.17%), *Enterococcus* (0.16%), and *Serratia* (0.15%). 

In traditional fermentation, *Lactobacillus* and *Acetobacter* had a relatively high abundance throughout the entire aged vinegar fermentation process, with *Lactobacillus* showing a downward trend in relative abundance and *Acetobacter* showing an increased trend. *Pediococcus* also had a relatively high abundance during the alcohol fermentation stage, reaching a maximum of 14.46%, which was an advantageous bacterial genus in traditional fermentation. *Pantoea*, *Weissella*, and *Bacillus* were detected during the early stages of alcohol fermentation, but their relative abundance was relatively low. As the fermentation time increased, their relative abundance decreased. In the acetic acid fermentation stage, the relative abundance of *Lactococcus* gradually increased, reaching 5.63% on the sixth day of acetic acid fermentation. Nie et al. employed denaturing gradient gel electrophoresis to investigate the dynamics and diversity of microbial community succession during the fermentation stage. The findings revealed that *Lactobacillus*, *Acetobacter*, *Yeasts*, and *Alternaria* emerged as the predominant microorganisms in the later phase of fermentation [26]. Wu et al. investigated the biodiversity of *lactic acid* bacteria during the fermentation process of Shanxi aged vinegar. The results revealed that *Lactobacillus fermentans*, *Lactobacillus plantarum*, *Lactobacillus casei*, and *Lactobacillus lactis* were the dominant microorganisms [2]. These results are consistent with those of previous studies [27,28]. 

### 3.3. Fermentation Characteristics of Selected Lactic Acid Bacteria L7, L19, L729, and L2422 from Shanxi Aged Vinegar in Different Culture Media

#### 3.3.1. Morphological Characteristics of *Lactic Acid* Bacteria L7, L19, L729, and L2422 in Different Media

**Figure 2:**
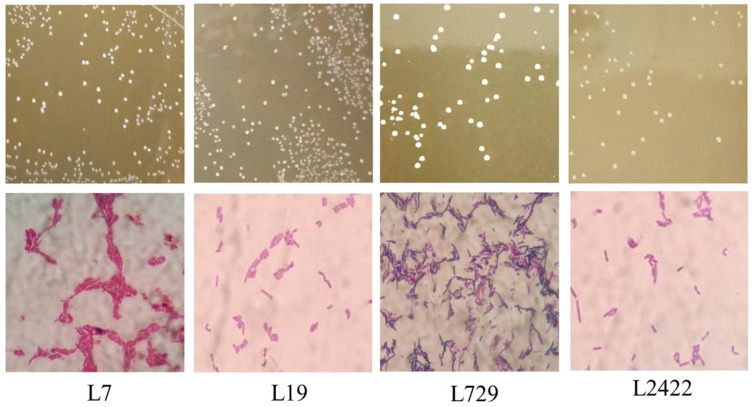
Colony morphology and cell morphology of *lactic acid* bacteria. Notes: L7: *Lactobacillus plantarum* SAVndL 7. L19: *Lactobacillus plantarum* SAVndL 19. L729: *Pediococcus acidilactici* SAVndL 729. L2422: *Pediococcus pentosaceus* SAVndL 2422.

Based on the preliminary research [2], four strains of *lactic acid* bacteria were isolated from the fermentation process of Shanxi aged vinegar, and their morphological characteristics were observed. The morphological characteristics of the *lactic acid* bacteria are presented in Table 1 and Figure 2.

#### 3.3.2. Study of the Growth Characteristics of Lactobacillus Strains L7, L19, L729, and L2422 in Different Media and the Characteristics of Total Acid Production, Reducing Sugar, and Acetoin Production

**Figure 3:**
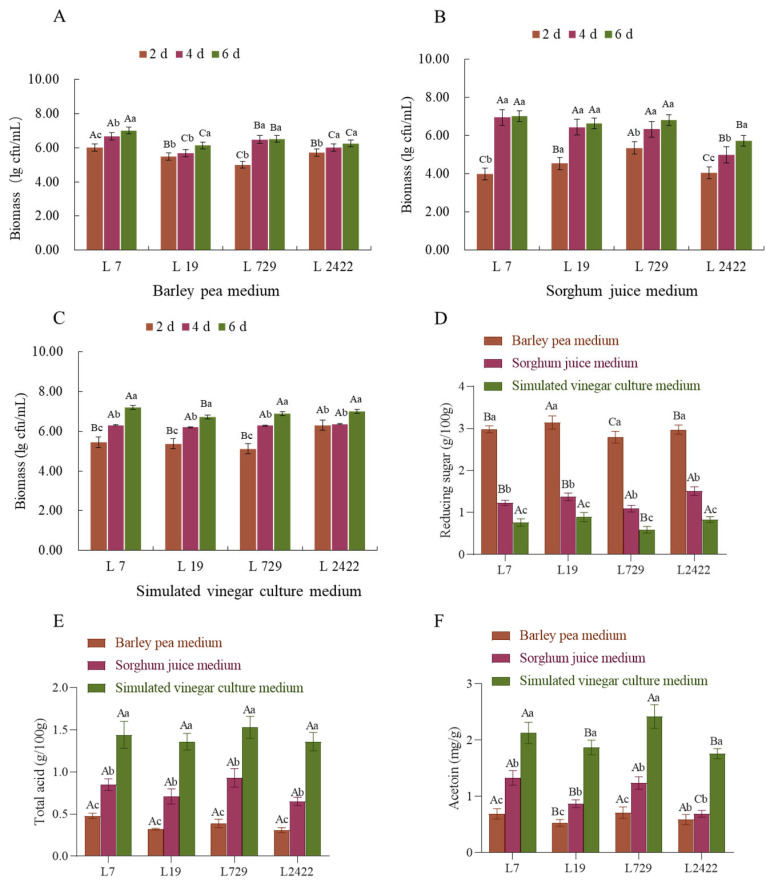
Growth characteristics of different *Lactobacillus* strains L7, L19, L729, and L2422 in different media and the characteristics of total acid production, reducing sugar, and acetoin production. Notes: (**A**) Biomass of *lactic acid* bacteria in barley and pea medium. (**B**) Biomass of *lactic acid* bacteria in sorghum juice medium. (**C**) The biomass of *lactic acid* bacteria in the fermented fermentation medium was simulated. (**D**) Reducing sugar utilization of *lactic acid* bacteria in different media. (**E**) Acid-producing properties of *lactic acid* bacteria in different media. (**F**) Acetoin production properties of *lactic acid* bacteria in different media. Capital letters represent changes in different strains in the same medium; lowercase letters represent changes in different media of the same strain.

The growth characteristics, total acid, reducing sugar, and acetoin production of strains L7, L19, L729, and L2422 were analyzed under the conditions of barley pea medium, sorghum juice medium, and simulated vinegar culture medium. As shown in Figure 3A, the biomass gradually increased with the increase in incubation time. In the barley pea medium, strain L7 exhibited the best growth by reaching a maximum biomass of 7.0 ± 0.18 log CFU/mL on day 6. As shown in Figure 3B, in the sorghum juice medium, strain L7 grew slowly and reached a maximum of 7.02 ± 0.21 log CFU/mL on the sixth day. As shown in Figure 3C, strain L7 exhibited the highest biomass of 7.20 ± 0.09 log CFU/mL on the sixth day in the simulated fermented vinegar medium, indicating its optimal growth. All four *lactic acid* bacteria tested showed a favorable growth trend in the simulated fermented vinegar medium. In Figure 3D, it can be observed that the content of reducing sugar in barley pea medium ranged from 2.79 ± 0.08 to 3.14 ± 0.13 g/100 g, which was relatively higher than that observed in other media. Furthermore, the strain L729 exhibited the lowest content of reducing sugar in both the sorghum juice medium and simulated vinegar culture medium. Figure 3E demonstrates that strains L7 and L729 exhibited a higher total acid production than did the other two *lactic acid* bacteria in all three media. Strain L7 achieved the highest acid yield of 0.48 ± 0.02 g/100 g in the barley pea medium. However, strain L729 exhibited the highest acid production when cultivated on the sorghum juice medium and the simulated vinegar culture medium. Additionally, we also investigated the changes in acetoin levels, which serve as a precursor for tetramethylpyrazine and enhance the flavor of Shanxi aged vinegar. As shown in Figure 3F, the barley pea medium exhibited the highest acetobutyric acid yield, with strain L729 having the highest content. In the sorghum juice medium, both strain L7 and strain L729 showed a high acetoin content. In the fermented vinegar culture medium, strains L7 and L729 had higher acetoin contents compared to the other two *lactic acid* bacteria mentioned earlier. The four *lactic acid* bacteria exhibited superior total acid production, lower reducing sugar content, and better acetoin production in the simulated fermented vinegar culture medium. Li et al. employed diverse aging techniques to investigate the physical and chemical properties as well as the temporal evolution of the flavor profile in Sichuan sun-dried vinegar. The experimental findings revealed that naturally aged vinegar exhibited a higher total acid content compared to vinegar subjected to constant temperature aging [29]. Wang et al. conducted the solid-state fermentation of vinegar using a rotary drum bioreactor (RDB) and conventional fermentation (TF) to investigate the biological activity and volatile aroma compounds during the fermentation process. The results confirmed that the levels of alcohol, reducing sugar, total acid, and amino nitrogen obtained through both fermentation methods exhibited similar trends. The total acid content in protovinegar fermented by RDB was 5.1% higher than that in TF, while the fermentation period was shortened by 6 days. Acetic acid and *lactic acid* were identified as the main organic acids detected, with increasing levels of total phenols, flavonoids, tetramethylpyrazine, acetoin, and diacetyl observed in RDB [30]. The change trend of this fermentation stage was consistent with that observed in highland barley vinegar [31] and Zhejiang rose vinegar [32].

#### 3.3.3. Study of the Characteristics of Organic Acids and Volatile Aroma Substances Produced by *Lactic Acid* Bacteria Strains L7, L19, L729, and L2422 in Different Media

**Figure 4:**
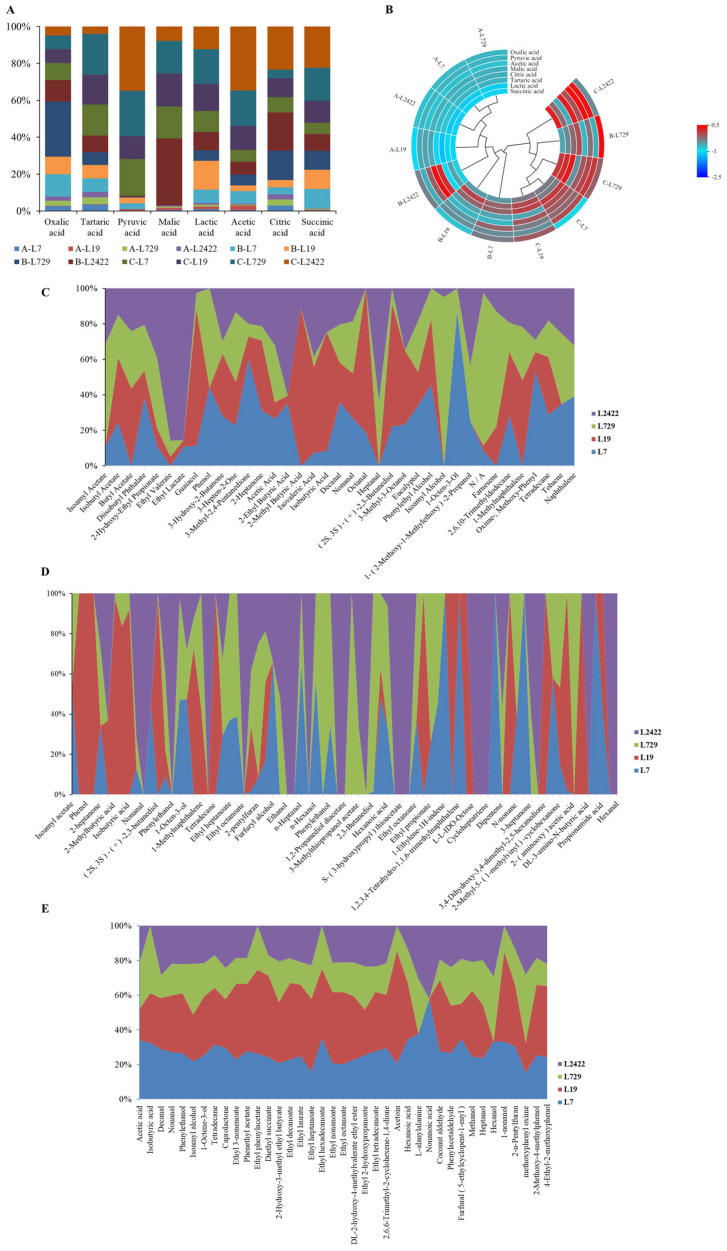
Characterization of organic acids and volatile aroma substances of *Lactobacillus* strains L7, L19, L729, and L2422 in different media. Notes: (**A**) The differences in organic acid content in different media. (**B**) Heat map of organic acid content in different media. (**C**) Aroma component content in barley and pea medium. (**D**) Aroma component content in sorghum juice medium. (**E**) Simulated the content of aroma components in simulated vinegar culture medium.

During the process of vinegar fermentation, a diverse array of microorganisms play a pivotal role in shaping its flavor profile through metabolic activities. These microorganisms enzymatically convert the nutrients present in the raw materials into volatile and non-volatile compounds such as alcohol, aldehyde, phenol, organic acid, reducing sugar, and amino acid derivatives, thereby imparting vinegar with its distinctive sensory attributes [33]. The dynamic changes in organic acid production of strains L7, L19, L729, and L2422 in different media were determined using high-performance liquid chromatography. Figure 4A,B display the results of the bar chart and the interaction ring heat map, indicating that strain L19 produced high levels of pyruvate, malic acid, *lactic acid*, acetic acid, and succinic acid in barley and pea medium. Among these acids, acetic acid content was 62.9 ± 35.2 mg/100 g. Strain L729 produced oxalic acid, tartaric acid, and citric acid with an oxalic acid content of 87.0 ± 3.6 mg/100 g. In the sorghum juice medium, strain L7 had the highest contents of pyruvate (27.2 ± 2.8 mg/100 g) and succinate (54.4 ± 4.2 mg/100 g). Strain L19 had the highest *lactic acid* content, while strain L729 had the highest oxalic acid content among all tested strains. Experimental evaluation allowed the identification of eight organic acids, including oxalic acid, tartaric acid, pyruvic acid, malic acid, *lactic acid*, acetic acid, citric acid, and succinic acid. We observed that acetic and *lactic acid*s were the main organic acids throughout the fermentation process. Overall, *lactic acid* bacteria produced varying amounts of organic acids in different media. This change tendency was consistent with the results of a previous study [16].

In addition, the dynamic changes in volatile aromatic substances in strains L7, L19, L729, and L2422 were also determined using gas chromatography in different media. The results shown in Figure 4C–E revealed that the fermentation products of barley pea medium, sorghum juice medium, and simulated vinegar culture medium yielded a total of 106, 90, and 100 volatile flavor compounds, respectively. In the barley and pea culture medium, strain L729 produced the highest total amount of esters, aldehydes, and alcohols (2.0136 ± 0.11 mg/L, 0.2770 ± 0.01 mg/L, and 3.7248 ± 0.09 mg/L, respectively). Strain L19 produced the highest amounts of phenols and ketones (1.1629 ± 0.08 mg/L and 5.1017 ± 0.13 mg/L, respectively). Strain L2422 produced the highest amount of acid (7.1863 ± 0.l7 mg/L). In the sorghum juice medium, strain L19 produced the highest amounts of alcohols, ketones, phenols, and acids with values of 3.1966 ± 0.07 mg/L, 6.8135 ± 0.18 mg/L, 1.2995 ± 0.06 mg/L, and 2.0352 ± 0.04 mg/L, respectively. Strain L729 produced the highest number of esters (3.6719 ± 0.11 mg/L), and strain L2422 produced the highest number of aldehydes (0.2431 ± 0.02 mg/L). Strain L7 exhibited the highest total production of aldehydes, alcohols, and acids in the simulated vinegar culture medium, with levels of 3.4971 ± 0.12 mg/L, 7.3465 ± 0.15 mg/L, and 6.1798 ± 0.18 mg/L, respectively. Strain L19 displayed the highest concentration of esters at 15.1445 ± 0.23 mg/L, while strain L2422 had the greatest content of ketones at 1.564 ± 0.07 mg/L. The contents of isoamyl alcohol, ethanol, and phenylethanol in the culture medium of simulated vinegar fermented grains were all greater than or equal to 1.2 ± 0.10 mg/L. These saturated fatty alcohols play an important role in contributing to the aroma of vinegar. The total amount of acids in the barley and pea medium was the highest, combined with the total amount of flavor substances in the fermentation products of the three different media. The content of ketones and phenols in the sorghum juice medium was the highest, while the total amount of esters, aldehydes, and alcohols was highest in the vinegar and fermented grains medium. It has been confirmed that the major metabolites produced by microorganisms exert a significant impact on the flavor characteristics [34]. However, there is a still limited understanding of the formation of volatile compounds in traditional vinegar production and their effects on the final product. Aromatic compounds have been predominantly identified as esters, followed by alcohols, aldehydes, and ketones [35,36]. For instance, an analysis of fermented grains from aged Shanxi aged vinegar revealed the presence of 26 esters, 6 alcohols, 5 aldehydes, and 4 ketones [26]. Zhang et al. identified a total of 35 volatile components from Zhejiang rose vinegar, which mainly consisted of alcohols, esters, acids, aldehydes, keto acids, phenols, and nitrogen-containing compounds [32]. These results of our experiment are similar to those of previous studies [16,35].

#### 3.3.4. Determination of Optimal *Lactic Acid* Bacteria Combination and Determination and Analysis of Biomass and Physicochemical Indicate in Pure Culture and Co-Culture Systems

**Figure 5:**
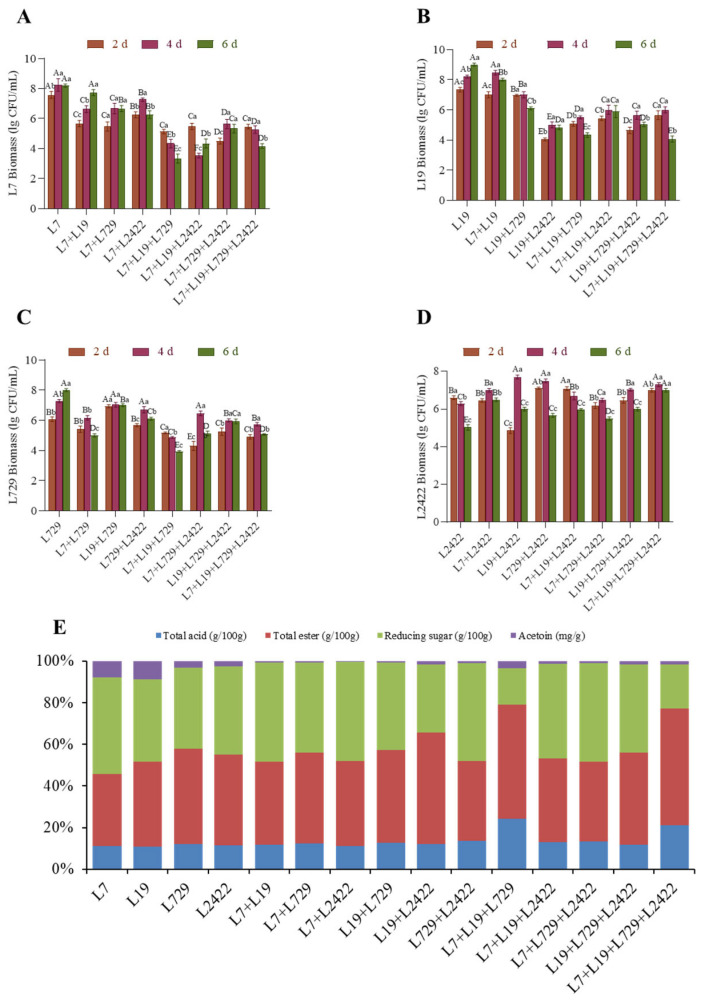
Biomass and physical and chemical indexes in pure culture and co-culture systems. Notes: (**A**) biomass of L7 in pure and co-culture systems; (**B**) biomass of L19 in pure and co-culture systems; (**C**) biomass of L729 in pure and co-culture systems; and (**D**) biomass of L2422 in pure and co-culture systems. (**E**) Changes in the content of sugars, total acids, total esters, and acetoin.

The biomass of L7, L19, L729, and L2422 strains in pure culture and co-culture systems was determined on a stimulated medium of vinegar-fermented grains. The results are shown in Figure 5. The biomass in the co-culture system was lower than that in the pure culture, which might be due to the mutual inhibition between *lactic acid* bacteria. As shown in Figure 5A, among the biomass of strain L7, the L19 + L729 system had the highest biomass with a value of 7.80 ± 0.19 log CFU/mL on the sixth day, representing an increase of approximately 8.3% compared to the pure culture. As shown in Figure 5B, among the biomass of strain L19, the biomass of the L7 + L19 system was the highest on the fourth day, reaching a maximum of 8.50 ± 0.21 log CFU/mL and decreasing to 8.0 ± 0.17 log CFU/mL on the sixth day. It is speculated that this may be due to the constant nutrient consumption, which limits bacterial growth. As shown in Figure 5C, among the biomass of strain L729, the biomass of the L19 + L729 co-culture was the highest, reaching 7.10 ± 0.11 log CFU/mL on the sixth day, which was 3.19% higher than that of the pure culture. As shown in Figure 5D, among the biomass of strain L2422, the L19 + L2422 co-culture exhibited superior biomass. The contents of reducing sugars, total acids, total esters, and acetyl proteins investigated in both pure and co-culture systems are depicted in Figure 5E. The reducing sugar content in the co-culture system was lower than that in the pure culture, but the difference was not significant. Compared to strains L7 and L729 in the pure culture, the acid production of strain L7 + L729 increased by 109.72% and 97.38%, respectively. In the co-culture system, except for strains L729 + L429 + L2422 and L7 + L19 + L2422, the total ester content of all other combinations was significantly higher than that of the pure culture. Compared to strains L7, L19, and L2422 in the pure culture, strain L719 + L19 + L2422 exhibited an increase in total ester content by 83.92%, 44.81%, and 57.25%, respectively. As a precursor of ligustrazine, acetoin can enhance the flavor of Shanxi aged vinegar [34]. Therefore, it is highly significant that we investigate the difference in acetoin content between the pure culture and co-culture systems for studying the interaction among different acetic acid strains. In addition, the content of acetoin in the co-culture system did not show a significant increase. The highest production of acetoin was observed when L7 + L729 were co-cultured in the system, resulting in a 13.14% increase compared to strain L7 in the pure culture. However, there was a slight decrease of 0.01 mg/g compared to strain L729 in the pure culture. This could be attributed to the inhibitory effect of strain L7 on strain L729, leading to a reduced biomass and acetoin production. It is crucial to determine the role of organic acids and acetoin in the production of Shanxi aged vinegar because they directly impact the flavor, nutritional value, and product quality of vinegar [37]. In terms of flavor formation, organic acids are key to creating the unique taste during acetic acid fermentation. Organic acids like acetic acid, malic acid, and tartaric acid not only provide vinegar with a distinct sour taste but also from various ester compounds through esterification with other components (such as alcohols), thereby enhancing the overall taste and flavor. Moreover, different ratios of organic acids can significantly influence the balance of flavors [38]. Acetoin serves as an important precursor for tetramethylpyrazine in Shanxi aged vinegar, playing a vital role in aroma development. In terms of nutritional value, organic acids and bioactive ingredients (e.g., acetic acid, gallic acid, catechin, epicatechin, chlorogenic acid, and caffeic acid) have significant functions in various physiological processes [38]. Firstly, they may lower gastrointestinal pH levels, which enhance mineral absorption by the body [39]. Secondly, the bacteriostatic and bactericidal properties possessed by organic acids can hinder harmful bacteria proliferation to maintain a healthy microbial balance [40]. Acetoin, as a precursor for functional components, may also be associated with producing certain bioactive substances that could positively affect health [37]. In terms of quality control, monitoring the content of organic acids in the fermented pastes allows us to ensure that the acetic acid fermentation process is carried out normally and avoid flavor deviations caused by excessive or insufficient fermentation. Simultaneously, determining the acetoin content can help screen and optimize fermentation strains, improve the yield of specific flavor products, and ensure product stability and consistency [41]. 

#### 3.3.5. Determination and Analysis of Organic Acid Content and Volatile Aroma Components in Pure Culture and Co-Culture Systems

**Figure 6:**
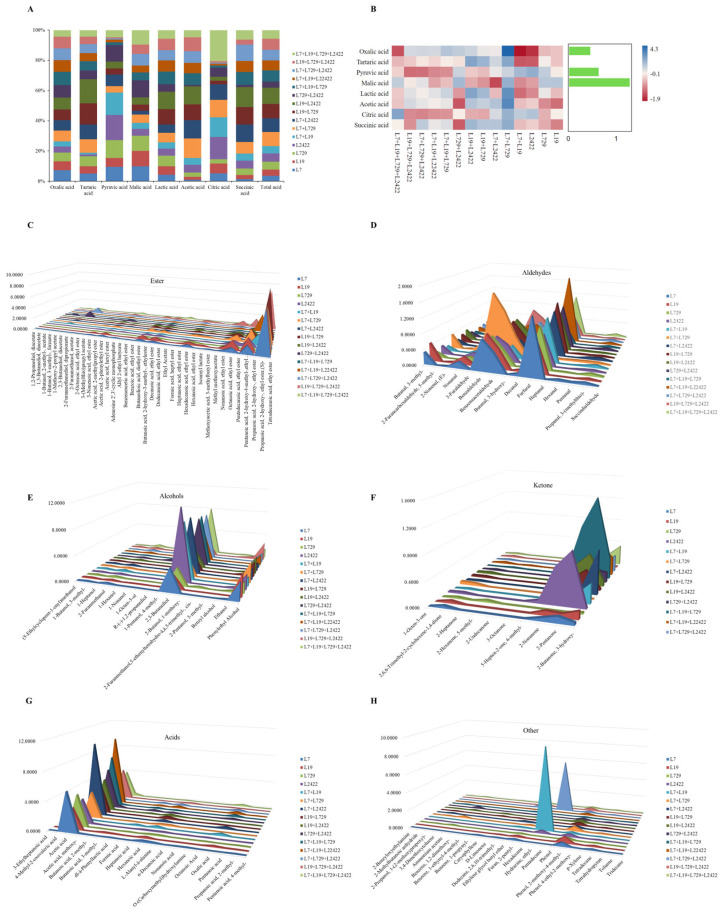
Determination and analysis of organic acid content and volatile aroma components in pure culture and co-culture systems. Notes: (**A**) Histogram analysis of organic acid content changes in pure culture and co-culture systems. (**B**) Heat map of organic acid content change in pure culture and co-culture system. (**C**) Changes in the contents of volatile aroma components esters in pure culture and co-culture systems. (**D**) Changes in the contents of volatile aroma components aldehydes in pure culture and co-culture systems. (**E**) Changes in the contents of volatile aroma components alcohols in pure culture and co-culture systems. (**F**) Changes in the contents of volatile aroma components ketones in pure culture and co-culture systems. (**G**) Changes in the acid content of volatile aroma components in pure culture and co-culture system. (**H**) Changes in other types of volatile aroma components in pure culture and co-culture systems.

In the acetic acid stage of Shanxi aged vinegar, acetic acid bacteria can efficiently oxidize small molecular sugars and ethanol, producing a large quantity of organic acids [9]. The content of organic acids was determined in both pure and co-culture systems, and the results are shown in Figure 6A,B. The total amount of organic acids in the pure culture and co-culture system ranged from 1.4287 ± 0.011 to 2.7293 ± 0.13 g/100 g, with the co-culture system having a higher total amount of organic acids than the pure culture. The total amount of organic acids in the co-culture system was highest for strain L19 + L2422, and, compared to strains L19 and L2422 in the pure culture system, the total amount of organic acids increased by 21.74% and 37.14%, respectively. Strain L7 + L19 + L729 had the highest oxalic acid content (0.3347 ± 0.06 g/100 g). The strain L7 + L19 + L729 + L2422 had the highest citric acid content, which was 0.2471 ± 0.02 g/100 g. In the co-culture system, the strain combination of L729 and L2422 exhibited the highest malic acid content (0.1802 ± 0.03 g/100 g). The strain combination of L7, L729, and L2422 had the highest succinic acid content at 0.2398 ± 0.01 g/100 g.

The co-culture and pure culture systems, as shown in Figure 6C–H, are mainly composed of acids, esters, and alcohols with a high content and diverse types. The results demonstrate that certain substances can only be produced in the co-culture system, such as ethyl phenylacetate, ethyl lactate, hexyl alcohol acetate, 5-methylfuranaldehyde, etc. Compared with strains L7 and L729 in the pure culture, the ester contents increased by 7 times and 12 times, respectively. The aldehyde substances also increased by 1.2 times and 1.6 times, respectively, compared to strains L7 and L729 in the pure culture. Additionally, the acid substances increased by 1.7 times and 2.3 times, respectively. The total amount of alcohol in the L7 + L2422 co-culture system was the highest, reaching 11.2925 ± 0.52 mg/L, which was four times higher than that in the pure culture and 3.2506 ± 0.13 mg/L lower than that in the pure culture. The total amount of ketones in the L7 + L19 + L729 co-culture system was the highest, measuring 2.6148 ± 0.19 mg/L. The above experimental results have confirmed that the co-culture system exhibited higher levels of organic acids and volatile aromatic compounds compared to the pure culture system, while variations in the content of these compounds were observed among different strains in the co-culture. In the fermentation process of Shanxi aged vinegar, the volatile aromatic compounds are mainly produced by various microorganisms such as *lactic acid* bacteria, yeast, acetic acid bacteria, and spore rods present during fermentation [26]. These compounds include esters, acids, alcohols, aldehydes, and other components with different contents that give Shanxi aged vinegar its unique flavor and provide it with a rich taste and aroma [42]. In addition to its main sour taste, vinegar also contains substances with a fruity aroma [42], floral aroma, and pleasant roasted and nutty flavors [43]. Slight changes in the types and contents of these flavor substances throughout the entire production process will affect the overall quality of vinegar. Zhou et al. employed gas chromatography—olfactometry—mass spectrometry, odor activity value, aroma recombination, and omission experiments to comprehensively characterize the key aroma compounds in aged Zhenjiang aromatic vinegar. In total, 68 compounds were quantified, with seven compounds identified as having the highest efficacy [44]. Li et al. confirmed that, in the traditional grain vinegar of Xinjiang, *lactic acid* and succinic acid were identified as the predominant organic acids alongside acetic acid. The elevated levels of acetoin, ethyl phenylacetate, ethyl phenylalcohol, and ethyl lactate contribute to the development of a delightful rosy, fruity, and creamy aroma during the later stages of aging [28]. These results of our experiment are similar to those of previous studies.

## 4. Conclusions

This study revealed the flora succession, physicochemical properties, and flavor substances of Shanxi aged vinegar at different fermentation stages. The fermentation characteristics of four *lactic acid* bacteria in different culture media were studied. The results showed that the biomass, physicochemical indexes, and flavor substances of four *lactic acid* bacteria in fermented vinegar medium were better than those in barley pea medium and sorghum juice medium. The changes of organic acids and volatile aroma components during the fermentation of Shanxi aged vinegar was further studied by pure culture and co-culture. Compared with the pure culture, the co-culture had a higher organic acid content and stronger volatile aroma. A total of 120 volatile aroma components and 8 organic acids were identified, among which esters were the main volatile compounds with rich aroma during Shanxi aged vinegar fermentation. Ethyl phenylacetate, ethyl lactate, and hexanol acetate were used as aroma biomarkers during fermentation, showing high odor activity. These findings will deepen our understanding of the physicochemical factors and flavor compounds during the fermentation process of Shanxi aged vinegar while helping to improve its quality.

## Figures and Tables

**Table 1:** The colony and cell morphology of *Lactic acid* bacteria in Shanxi aged vinegar.

Bacterial Strain	Fermentation Characteristics	Tolerance Property	Cell Morphology	Colonial Morphology
*Lactobacillus plantarum* SAVndL 7	High acid production (24.30 g/L), high acetoin production (1.21 mg/mL).	Alcohol (10%), high-temperature-resistant (50 °C), the initial sugar (250 g/L)	Slender, short rod	Colony diameter 1 mm, round, flat, smooth surface, shiny, neat edges, milky white, opaque.
*Lactobacillusplantarum* SAVndL 19	Acid production (21.95 g/L), acetoin content (1.09 mg/mL)	Alcohol (10%), high-temperature-resistant (50 °C), the initial sugar (250 g/L)	Slender, short rod	Colony diameter 0.8 mm, round, flat, smooth surface, shiny, neat edges, milky white, opaque.
*Pediococcus acidilactici* SAVndL 729	Acid production (18.55 g/L), acetoin content (0.92 mg/mL)	Alcohol (10%), high-temperature-resistant (50 °C), the initial sugar (250 g/L)	Rod shape	Colony diameter 1.2 mm, round, slightly convex, smooth surface, glossy, edge.
*Pediococcus pentosaceus* SAVndL 2422	Acid production (10.08 g/L), acetoin content (0.90 mg/mL)	Alcohol (8%), high-temperature-resistant (45 °C), the initial sugar (200 g/L)	Rod shape	Colony diameter 1 mm, round, raised, smooth surface, shiny, neat edges, milky white, opaque.

## Data Availability

The original contributions presented in the study are included in the article, further inquiries can be directed to the corresponding author.
